# Refining the Role of 5-HT in Postnatal Development of Brain Circuits

**DOI:** 10.3389/fncel.2017.00139

**Published:** 2017-05-23

**Authors:** Anne Teissier, Mariano Soiza-Reilly, Patricia Gaspar

**Affiliations:** ^1^Institut du Fer à Moulin, Institut National de la Santé et de la Recherche Médicale (INSERM), UMR-S839Paris, France; ^2^Université Pierre et Marie CurieParis, France; ^3^Institut du Fer à MoulinParis, France

**Keywords:** serotonin transporter, cortex, interneurons, tryptophan hydroxylase, mouse, fluoxetine

## Abstract

Changing serotonin (5-hydroxytryptamine, 5-HT) brain levels during critical periods in development has long-lasting effects on brain function, particularly on later anxiety/depression-related behaviors in adulthood. A large part of the known developmental effects of 5-HT occur during critical periods of postnatal life, when activity-dependent mechanisms remodel neural circuits. This was first demonstrated for the maturation of sensory brain maps in the barrel cortex and the visual system. More recently this has been extended to the 5-HT raphe circuits themselves and to limbic circuits. Recent studies overviewed here used new genetic models in mice and rats and combined physiological and structural approaches to provide new insights on the cellular and molecular mechanisms controlled by 5-HT during late stages of neural circuit maturation in the raphe projections, the somatosensory cortex and the visual system. Similar mechanisms appear to be also involved in the maturation of limbic circuits such as prefrontal circuits. The latter are of particular relevance to understand the impact of transient 5-HT dysfunction during postnatal life on psychiatric illnesses and emotional disorders in adult life.

## Introduction

Among the multifaceted roles serotonin (5-hydroxytryptamine, 5-HT) in the brain, its developmental role remains one of the most intriguing and challenging. A main interest for this question was triggered by the realization that one of the primary roles of 5-HT in psychiatric disorders, such as autism or anxiety-related disorders could have a developmental origin (Gaspar et al., 2003; Gross and Hen, 2004; Bonnin and Levitt, 2011; Dayer, 2014). Furthermore, the long delay required for antidepressant action suggested that the positive effects of drugs enhancing 5-HT transmission could involve the re-activation of developmental plasticity mechanisms (Tiraboschi et al., 2013). However, understanding the underlying cellular and molecular mechanisms remains challenging, in part due to the existence of 14 different receptor subtypes that can all trigger trophic signals and that display a highly dynamic developmental expression pattern. Furthermore, 5-HT is known to influence every single developmental process from neurogenesis, cell migration, axon guidance, dendritogenesis, to synaptogenesis (Lauder et al., 1981; Gaspar et al., 2003; Sodhi and Sanders-Bush, 2004; Daubert and Condron, 2010; Trakhtenberg and Goldberg, 2012). Thus, 5-HT’s “developmental” effects and its underlying cellular/molecular mechanisms are likely to vary across lifespan.

In this minireview article, we chose to focus on postnatal development, when activity-dependent mechanisms sculpt neural circuits in response to the environment. This plasticity is maximal during early postnatal life in rodents, coinciding with high levels of 5-HT in the brain. However similar mechanisms persist to some extent in adult life and can be reactivated by increasing brain levels of 5-HT. A handful of neural systems allowed a clear demonstration of 5-HT’s actions on structural fine-tuning of neural circuits. One is the 5-HT raphe system, raising the question of feedback control of 5-HT on its own maturation. The other model systems are the sensory systems (somatosensory and visual) that have the advantage of well-defined topographic organizations. We summarize here some recent genetic/pharmacological evidence that provide new insights on the mechanisms involved in 5-HT’s developmental effects and on the role of 5-HT in promoting adult neural plasticity.

## Role of 5-HT on the Maturation of Raphe Circuits

### 5-HT Autocrine Effects on Axonal Growth Relies on Local Cues

The notion that 5-HT controls its own development by autocrine mechanisms stems from the identification of 5-HT inhibitory autoreceptors on raphe neurons. This led to suggest that autoreceptors control the production and differentiation of 5-HT neurons during development (De Vitry et al., 1986; Whitaker-Azmitia and Azmitia, 1986; Liu and Lauder, 1991). Raphe 5-HT neurons are born early during development (E10–E12 in rodents, GW 4–5 in humans) but maturation of 5-HT innervation is protracted during post-natal life (P21 in rodents; Olson et al., 1973; Gaspar et al., 2003). Observations in knock-out mice for the 5-HT transporter (SERT-KO) and in rodents receiving 5-HT selective reuptake inhibitors (SSRIs) during post-natal development suggested that increasing brain 5-HT levels could hamper the maturation of the 5-HT raphe neurons, reducing the number of neurons and the density of terminal innervation (Lira et al., 2003; Silva et al., 2010; Weaver et al., 2010). However this was not supported by observations in other genetic mouse strains with increased brain 5-HT levels (Cases et al., 1995). Furthermore, in 5-HT deficient mice, no difference in the number of 5-HT raphe neurons or in the density of SERT-positive terminal innervations was found (Gutknecht et al., 2008; Narboux-Nême et al., 2013). Invertebrate models also showed that 5-HT neurons develop normally in the absence of monoamine production (Sze et al., 2000). These conflicting observations led to question the autocrine role of 5-HT.

**Figure 1:**
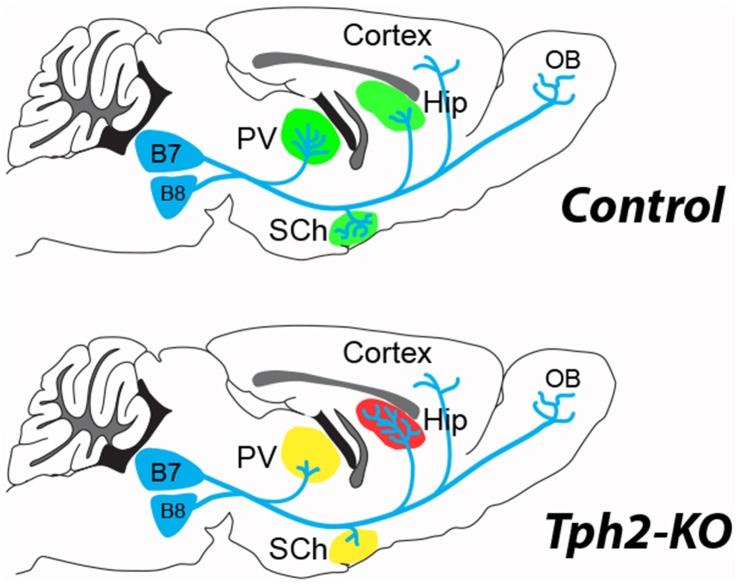
**5-hydroxytryptamine (5-HT)-depletion in the tryptophan hydroxylase 2 (Tph2)-KO mouse modifies the terminal innervation of 5-HT neurons from dorsal raphe, B7 and median raphe, B8 (Migliarini et al., 2013).** The green shading shown in the control brain, corresponds to areas where modified 5-HT innervation was observed in the Tph2-KO mice (red, increased; yellow, decreased). An increased density of 5-HT terminals (red shading) was noted in the hippocampus (Hip) coinciding with a decreased innervation (yellow shading) of the thalamic paraventricular (PV) nucleus and the suprachiasmatic (SCh) nucleus. No difference in 5-HT fiber density was observed in the cortex or the olfactory bulb (OB) of the Tph2-KO. Similar observations were made after adult knockdown of Tph2 (Pratelli et al., 2017).

New evidence on the structural organization of raphe neurons in 5-HT-deficient mice clarified this issue. Pasqualetti and colleagues used GFP reporter expression in the Tryptophan hydroxylase 2 (Tph2) gene locus to fate-map 5-HT–raphe neurons in the absence of central 5-HT (Migliarini et al., 2013). Their results demonstrate striking region-specific modifications of the raphe fibers. In the absence of 5-HT, raphe neurons are produced and survive in normal numbers but defects in the density of innervations arise during the late phases of axon targeting and remodeling (Figure 1). In a more recent study they investigated the role of 5-HT transmission in maintaining adult raphe circuits (Pratelli et al., 2017); 3 weeks after ablation of 5-HT synthesis similar defects in 5-HT innervation were observed. These results suggest the establishment and maintenance of serotonergic terminal innervation both rely on local 5-HT signaling in brain targets rather than on a cell-autonomous role of 5-HT on its own maturation.

These observations also emphasize the potential of 5-HT raphe axons to remodel during the entire life corroborating observations in adults. The capacity of 5-HT axons to regenerate and sprout after adult lesions has been widely demonstrated after different types of lesions. For instance lesions of the dopaminergic neurons lead to compensatory 5-HT sprouting in the striatum (Descarries et al., 1992; Gaspar et al., 1993; Gagnon et al., 2016). A rapid growth of 5-HT axons after spinal cord injury (either traumatic or ischemic) has also been repeatedly demonstrated (von Euler et al., 2002; Müllner et al., 2008). The unusual response of serotonergic neurons after CNS injury could be due to a lack of axonal dieback and enhanced sprouting in the environment of the glial scars (Hawthorne et al., 2011). However recent *in vivo* imaging data showed that cut 5-HT axons undergo an initial regression process followed by a regrowth that does not follow pre-existing axon tracts (Jin et al., 2016). Further studies should identify the local cues that are controlled by 5-HT and are responsible for attraction/repulsion of 5-HT fibers in select targets. One interesting developmental molecular correlate is BDNF whose expression is up-regulated in the hippocampus of constitutive Tph2–KO (Migliarini et al., 2013). A role of 5-HT in the expression of local guidance molecules would be interesting to explore further since specific guidance molecules such as ephrinAs have recently been shown to direct the axonal growth of selected subpopulations of raphe neurons to defined brain targets in the hypothalamus and amygdala (Teng et al., 2017).

### Control of Raphe Neuron Excitability by Changes in Neurotransmission Signaling

Recent work revealed important features in the postnatal maturation of rodent raphe neurons (Rood et al., 2014; Morton et al., 2016). These studies described the progressive arrival of excitatory and inhibitory inputs on 5-HT and GABA raphe neurons from P4 to P21, in correlation with the maturation of electrophysiological firing properties, including the appearance of 5-HT1A auto-receptor responses (Rood et al., 2014). This work also revealed that most of the electrophysiological diversity previously described between raphe nuclei emerges during this postnatal time window (Rood et al., 2014). Hence, these observations suggest that the differential maturation of 5-HT1AR signaling and differences in inputs could both contribute to 5-HT neurons physiological diversity (Calizo et al., 2011; Kiyasova et al., 2013; Fernandez et al., 2016). Interestingly, exposure of mouse pups (P2–P21) to SSRIs permanently impaired neuronal firing of raphe neurons with opposite changes in the dorsal vs. the medial nuclei (Teissier et al., 2015). These results suggest a different sensitivity of 5-HT neurons and/or of their inputs to increased levels of 5-HT during postnatal development. Accordingly, transient silencing of 5-HT1AR expression in 5-HT neurons (P14–P30) also caused increased excitability of the dorsal raphe (Donaldson et al., 2014) suggesting a cell-autonomous mechanism. However, it is also possible that the strength and/or the density of the inputs is modified by developmental 5-HT. Given the effects of adult stress on the excitability of raphe neurons (Crawford et al., 2013; Challis and Berton, 2015) high degree of plasticity is maintained in raphe circuits, notably via the control of local raphe interneurons activity and changes in expression of the calcium-activated potassium channels (Sargin et al., 2016).

In the perspective of identifying molecular candidates that control raphe neurons development, trancriptome analyses were done on sorted 5-HT neurons at embryonic (E11–E15) and early postnatal stages (P2; Wylie et al., 2010; Wyler et al., 2016). These experiments identified a large number of receptors, including receptors for glutamate, acetylcholine, GABA, cannabinoids, glucocorticoids and estrogens all of which are potential targets for input-dependent developmental effects. Accordingly, studies have pointed to developmental impact of nicotine receptor on 5-HT neuron excitability (Cerpa et al., 2015).

## New Insights on the Role of 5-HT on Somatosensory Map Maturation

The developmental role of 5-HT in the refinement of circuits has been best documented in the construction of sensory maps. The clear topographic organization of sensory afferents in the cortex and subcortical relays made them ideal models to analyze neurodevelopmental mechanisms in general and the role of 5-HT in particular (Erzurumlu and Gaspar, 2012; Assali et al., 2014).

### 5-HT Induced Changes of Cortical Microcircuits in the Barrel Cortex

**Figure 2:**
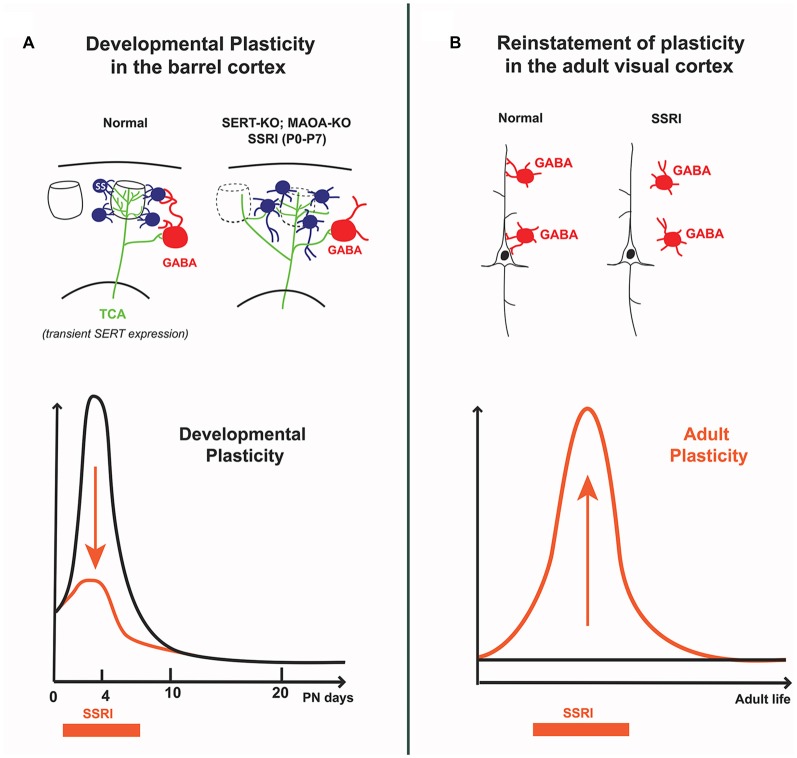
**Differential effects of 5-HT on cortical circuits assembly and plasticity in early postnatal vs. adult life. (A)** In the developing barrel cortex, transient excess of 5-HT (i.e., SERT-KO, MAOA-KO and 5-HT selective reuptake inhibitors (SSRI) exposure from P0 to P7) alters the “barrel map” circuit organization; thalamocortical axons extend across different barrel domains and spiny stellate neurons in layer 4 do not orient dendrites toward barrel centers, furthermore their axons abnormally extend into the lower cortical layers. Decreased feed-forward inhibition is also observed, indicating that both excitatory and inhibitory cortical circuits are modified by early exposure to 5-HT. **(B)** In the adult visual cortex, SSRI exposure increases 5-HT levels and reinstates ocular dominance (OD) plasticity which is normally absent at this stage; this involves a reduction in the inhibitory control of cortical pyramidal neurons by local GABA interneurons. The curves schematize the different effects of SSRIs at different life periods: during early postnatal life SSRIs dampen developmental plasticity, whereas in adults SSRIs increase cortical plasticity.

In the rodent somatosensory cortex, the so-called “barrel cortex”, early studies showed that brief modifications of 5-HT signaling during early postnatal development of rodents had striking effects on the organization of the barrels (Erzurumlu and Gaspar, 2012; van Kleef et al., 2012). Increasing 5-HT levels during the first postnatal week was sufficient to perturb the clustering of thalamocortical axons (TCAs) and the organization of layer 4 neurons into columnar, periphery-related patterns in the cerebral cortex. At a finer level, single neuron reconstruction studies showed that this was due to defective terminal branching of TCAs and to a failure of dendritic orientation towards barrel centers in layer 4 neurons (Rebsam et al., 2002; Lee et al., 2009; Figure 2A).

Recent detailed anatomical and physiological studies provided a full description of the local cortical architecture relying on 5-HT for its development. In SERT-KO rats, Miceli et al. (2013) showed that not only were TCAs exuberantly crossing though several columns, but that the excitatory neurons in layer 4 (spiny stellate and pyramidal neurons), lost their intracolumnar axonal projections with increased output projections to infragranular layer 5b (Miceli et al., 2013). In addition, they found that local inhibitory circuits were altered, with reduced feed-forward inhibition and reduced perisomatic inhibitory synapses and suggested that part of this effect could be due to a modified expression of the KCC2 chloride transporter (Miceli et al., 2017; Figure 2A). Overall, these findings indicate that the postnatal effects of 5-HT on the organization and function of the barrel cortex have greater impact on intracortical circuit maturation than previously thought, and that different 5-HT-dependent mechanisms could be at play in the different cellular elements of the barrel cortex.

### 5-HT Inhibits Somatosensory Cortical Input during Critical Periods

Sensory inputs that rely on glutamatergic transmission, have an instructive role for barrel formation (Erzurumlu and Gaspar, 2012). Genetic and pharmacological evidence indicated that 5-HT signaling controls TCA glutamatergic inputs via presynaptic 5-HT1B receptors that are transiently expressed in thalamic neurons (Bennett-Clarke et al., 1993). Strikingly, invalidation of the 5-HT1BR gene rescued the barrelless phenotype of monoamine oxidase A-KO and SERT-KO mice (Salichon et al., 2001). 5-HT1BRs have a dual role as they negatively control excitatory thalamocortical synapses (Rhoades et al., 1994; Laurent et al., 2002), and promote axonal growth (Bonnin et al., 2007). The main role of 5-HT as a modulator of cortical activity was recently stressed by *in vivo* electrophysiological studies in rat pups. Akhmetshina et al. (2016) increased 5-HT brain levels by administering an SSRI, citalopram and showed that this resulted in a rapid suppression of spontaneous cortical activity in the somatosensory cortex and inhibited sensory-evoked oscillatory bursts (Akhmetshina et al., 2016). Interestingly, such effects were not observed when citalopram was administered in adolescence.

Another line of evidence suggested a different type of interaction between sensory afferents and 5-HT signaling in barrel cortex development, indicating that birth plays a major role in the formation of the barrels via changes in 5-HT transmission (Toda et al., 2013). Toda et al. (2013) showed that barrel development was accelerated in pups with preterm birth and identified 5-HT signaling as a key downstream mediator in this mechanism. Specifically, they showed that soon after birth the extracellular levels of 5-HT were dramatically reduced and that this could trigger the initiation of barrel formation. It is not quite clear how birth induces a reduction in 5-HT levels. The placenta is an extra-embryonic source of 5-HT that is arrested at birth. However, placental 5-HT contributed to only a negligible source after E15 (Bonnin et al., 2011). Toda et al. (2013) suggest that this decrease could result from an increased local uptake/degradation of 5-HT rather than a decreased 5-HT synthesis. Consistent with this idea, SERT expression increased both in sensory thalamic and raphe neurons soon after birth (Lebrand et al., 1998), and accordingly, local application of SERT blockers inhibits the precocious barrel formation in preterm pups. Application of a non-selective 5-HT1 antagonist (but not of a specific 5-HT1BR antagonist) was able to induce a precocious barrel formation, indicating that other members of the 5-HT1 receptor subfamily would play a role in this developmental mechanism (Toda et al., 2013).

### Cell-Autonomous Role of SERT in Thalamocortical Neurons

Transient expression of the SERT has been suspected to play a role in barrel cortex and visual map development, since SERT is expressed in the sensory thalamic relay neurons, and retina, during the critical periods of sensory map development (Gaspar et al., 2003; Homberg et al., 2010). The broad extra-raphe SERT expression in glutamatergic neurons was initially suggested to allow 5-HT to act as a borrowed neurotransmitter, however this hypothesis was not supported in hyposerotonergic mouse models where the barrel cortex develops normally, showing that 5-HT is dispensable (van Kleef et al., 2012; Narboux-Nême et al., 2013). Conversely, transient SERT expression could serve to uptake, and degrade extracellular 5-HT, preventing excessive local activation of 5-HT1BRs located in the same axon terminals (Salichon et al., 2001). Recently, a very elegant genetic study in mouse allowed to test specifically the role of SERT expression in glutamatergic neurons during development (Chen et al., 2015). Using a conditional genetic approach, Chen et al. (2015) showed that deleting SERT expression in the Vglut2-glutamatergic neurons, including thalamocortical neurons, prevented the normal refinement of TCAs, and layer 4 barrel organization, whereas deleting SERT from the brainstem 5-HT neurons had no visible effects. Additionally, they showed that deleting SERT expression from the cortical pyramidal neurons had no effect on TCA patterning (Chen et al., 2016), although other possible effects on cortical microcircuits (Miceli et al., 2013, 2017) were not investigated. Overall, these results stress a likely clearance role for transient SERT expression in non-serotonergic glutamate neurons during development. Interestingly, similar local 5-HT scavenging has been described in *Caenorhabditis elegans* where 5-HT is taken up by 5-HT-absorbing neurons, that prevent excessive 5-HT signals at extrasynaptic targets in a behavioral neural circuit (Jafari et al., 2011).

## Role of 5-HT in Visual Cortex Plasticity: Ocular Dominance and Cross-Modal Plasticity

The capacity of 5-HT to promote cortical plasticity was initially demonstrated during the critical period of ocular dominance (OD) plasticity in kittens (P20–P40). During this period transient regional and columnar specific organization of 5-HT2 receptors were noted, that were dependent on sensory visual inputs (Dyck and Cynader, 1993) and 5-HT2C antagonists reduced OD plasticity (Gu and Singer, 1995; Wang et al., 1997). During an equivalent critical period in rodents, 5-HT was shown to control cross-modal plasticity between the visual and somatosensory cortex; this involved 5-HT2A/2C receptor-dependent synaptic mechanisms (Jitsuki et al., 2011). Visual deprivation in juvenile rats (P21 to P23) increased extracellular 5-HT levels in the barrel cortex, by an undetermined mechanism. This resulted in a facilitation of synaptic strength in the barrel cortex after whisker stimulation. Overall, this experiment indicated that 5-HT signaling could be an effector of activity-dependent competitive interaction between sensory areas.

Remarkably, OD developmental plasticity can be reinstated in adult rats by raising 5-HT levels, notably using SSRIs (Maya Vetencourt et al., 2008; Vetencourt et al., 2011; Figure 2). Similarly, environmental enrichment restored OD plasticity through a 5-HT-dependent mechanism (Baroncelli et al., 2010; Balog et al., 2014). Reinstatement of OD plasticity in adult cortex was accompanied by improved recovery of adult visual acuity in particular after eye closure, leading to propose fluoxetine as a treatment of amblyopia. The underlying mechanisms were found to involve a local disinhibitory network resulting from decreased activity of GABAergic interneurons and increased BDNF expression (Sale et al., 2007; Chen et al., 2011). In a recent study analyzing protein synaptic changes, more mature glutamate and GABA subunit receptors were found rather than the expected shift toward immature patterns (Beshara et al., 2016). Therefore, precise synaptic mechanisms remain to be identified.

These observations in the visual system have incited researchers to investigate whether 5-HT agonists such as antidepressants could improve adult plasticity in a variety of other pathological conditions. In a model of spinal cord injury, administration of 5-HT1AR agonists enhanced cortical reorganization of the sensorimotor cortex, increasing the representation of the intact forelimb and improving behavioral performance (Ganzer et al., 2013). In a mouse model of stroke, fluoxetine administration promoted cortical plasticity and behavioral recovery by decreasing the drive of local inhibitory parvalbumin interneurons (Ng et al., 2015). This mechanism is consistent with the observations after adult OD plasticity reinstatement (Sale et al., 2007; Maya Vetencourt et al., 2008; Chen et al., 2011).

## Perspectives: Role of 5-HT in The Maturation of Limbic Circuits Underlying Emotional Disorders

This overview highlights the role of 5-HT during critical periods of plasticity for the maturation of neural circuits, and start uncovering the neurobiological bases of 5-HT’s role in neurodevelopmental disorders such as autism, schizophrenia, or anxiety disorders (Suri et al., 2014; Marín, 2016). The key role of 5-HT dysfunctions in psychiatric disorders has received further support by experiments showing that manipulations of 5-HT signaling during development or in adulthood can either prevent or rescue behavioral disorders in genetic mouse models of autism or in stress-induced models of emotional disorders (Benekareddy et al., 2011; Sarkar et al., 2013; Teissier et al., 2015; Luo et al., 2017). However, critical periods of vulnerability during the development of limbic system still need to be formally demonstrated at a circuit level. The prefrontal cortex is a primary suspect in that regard. Altered structural and functional maturation of the PFC neurons was demonstrated in SERT-KO mice (Wellman et al., 2007) and after early SSRI administration (Rebello et al., 2014). However, the precise PFC circuits impacting mood behavior after postnatal or adolescent perturbations of 5-HT signaling (Gross et al., 2002; Alexandre et al., 2006; Sarkar et al., 2013; Goodfellow et al., 2014; Soiza-Reilly et al., 2015; Garcia-Garcia et al., 2017) remain unclear, and neural circuits interconnecting the PFC with the amygdala or with brainstem 5-HT neurons could play a role. Altered PFC-amygdala pathway was noted in hyposerotoninergic mice (Narayanan et al., 2011; Dzirasa et al., 2013), and PFC-raphe circuit was involved in the control of stress responses (Warden et al., 2012; Challis and Berton, 2015). Future research should allow determining whether and how the assembly and maturation of these circuits is controlled by 5-HT during critical periods of early postnatal life.

## Author Contributions

All authors collected references, wrote the manuscript and prepared figures.

## Funding

Research in the Gaspar laboratory is supported by the Fondation de la Recherche Médicale and the Agence Nationale de la Recherche (ANR-11-0004-02; ANR-15-0179, ANR-16-0162), the INSERM, and Université Pierre et Marie Curie. The team is part of the Ecole des Neurosciences de Paris training network supported by the Investissements d’Avenir program, managed by the ANR under the reference ANR-11-IDEX-0004-02.

## Conflict of Interest Statement

The authors declare that the research was conducted in the absence of any commercial or financial relationships that could be construed as a potential conflict of interest.
